# ^18^F-BMS986192 PET Imaging of PD-L1 in Metastatic Melanoma Patients with Brain Metastases Treated with Immune Checkpoint Inhibitors: A Pilot Study

**DOI:** 10.2967/jnumed.121.262368

**Published:** 2022-06

**Authors:** Pieter H. Nienhuis, Inês F. Antunes, Andor W.J.M. Glaudemans, Mathilde Jalving, David Leung, Walter Noordzij, Riemer H.J.A. Slart, Erik F.J. de Vries, Geke A.P. Hospers

**Affiliations:** 1 Department of Nuclear Medicine and Molecular Imaging, Medical Imaging Center, University of Groningen, University Medical Center Groningen, Groningen, The Netherlands;; 2 Department of Medical Oncology, University of Groningen, University Medical Center Groningen, Groningen, The Netherlands;; 3 Bristol Myers Squibb, Princeton, New Jersey; and; 4 Faculty of Science and Technology, Department of Biomedical Photonic Imaging, University of Twente, Enschede, The Netherlands

**Keywords:** PET, PD-L1 expression, metastatic melanoma, brain metastasis, immune checkpoint inhibition

## Abstract

Immune checkpoint inhibitors (ICIs) targeting programmed cell death protein-1 (PD-1)/programmed cell death ligand-1 (PD-L1) frequently induces tumor response in metastatic melanoma patients. However, tumor response often takes months and may be heterogeneous. Consequently, additional local treatment for nonresponsive metastases may be needed, especially in the case of brain metastases. Noninvasive imaging may allow the characterization of (brain) metastases to predict response. This pilot study uses ^18^F-BMS986192 PET for PD-L1 expression to explore the variability in metastatic tracer uptake and its relation to tumor response, with a special focus on brain metastases. **Methods:** Metastatic melanoma patients underwent whole-body ^18^F-BMS986192 PET/CT scanning before and 6 wk after starting ICI therapy. ^18^F-BMS986192 uptake was measured in healthy tissues, organs, and tumor lesions. Tumor response was evaluated at 12 wk using CT of the thorax/abdomen and MRI of the brain. RECIST, version 1.1, was used to define therapy response per patient. Response per lesion was measured by the percentage change in lesion diameter. Toxicity was assessed according to Common Terminology Criteria for Adverse Events, version 4.0. **Results:** Baseline ^18^F-BMS986192 PET/CT was performed in 8 patients, with follow-up scans in 4 patients. The highest tracer uptake was observed in the spleen, bone marrow, kidneys, and liver. Tracer uptake in tumor lesions was heterogeneous. In total, 42 tumor lesions were identified at baseline, with most lesions in the lungs (*n* = 21) and brain (*n* = 14). Tracer uptake was similar between tumor locations. ^18^F-BMS986192 uptake in lesions at baseline, corrected for blood-pool activity, was negatively correlated with the change lesion diameter at response evaluation (r = −0.49, *P* = 0.005), both in intra- and extracerebral lesions. Receiver-operating-characteristic analysis demonstrated that ^18^F-BMS986192 uptake can discriminate between responding and nonresponding lesions with an area under the curve of 0.82. At the follow-up scan, an increased ^18^F-BMS986192 uptake compared with baseline scan was correlated with an increased lesion diameter at response evaluation. In the follow-up ^18^F-BMS986192 PET scan of 2 patients, ICI-related toxicity (thyroiditis and colitis) was detected. **Conclusion:** In this pilot study, ^18^F-BMS986192 PET showed heterogeneous uptake in intra- and extracerebral metastatic lesions in melanoma patients. Baseline ^18^F-BMS986192 uptake was able to predict an ICI treatment–induced reduction in lesion volume, whereas the follow-up PET scan allowed the detection of treatment-induced toxicity.

## Visual Abstract

**Figure:**
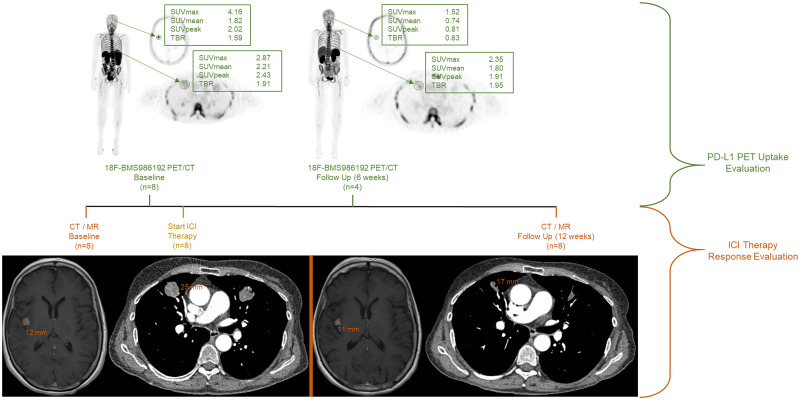

Immune checkpoint inhibitors (ICIs) targeting the programmed cell death protein-1 (PD-1)/programmed cell death ligand-1 (PD-L1) axis or cytotoxic T-lymphocyte–associated protein 4 (CTLA-4) receptor, either as monotherapy or as combined treatment, have shown response rates of approximately 40%–60% in patients with metastatic melanoma, with progression-free and overall survival (*1*–*8*).

However, it often takes months before response to ICI treatment can be detected, and lesions may respond in a heterogeneous manner. Some lesions may respond, whereas other lesions may not respond or display pseudoprogression (*9*). Consequently, some fast-growing lesions may require additional treatment. Data from the Dutch National Cancer Registry show that 22% (876/3,959) of metastatic melanoma patients underwent surgery for nonresponding metastases in addition to systemic treatment with ICI (*10*). Moreover, approximately 30% of metastatic melanoma patients develop brain metastases. Brain metastases are considered as a risky location because they carry a high risk for developing central nervous system failure and mortality (*11*). Therefore, local treatment with radiotherapy or resection is often considered in patients with brain metastases.

Before starting ICI therapy, it is difficult to determine which lesions will respond and which lesions will require additional treatment. Immunohistochemistry on biopsy samples only provides information about a small fraction of a single tumor lesion and is, therefore, unable to address intra- and intertumoral heterogeneity, thus limiting the value of immunohistochemistry to predict response. In patients with metastatic melanoma, known to be a high heterogenic responding tumor, PD-1 immunohistochemistry staining on biopsies was not a good predictor of response to ICI and hence is not used in clinical practice (*12*).

Whole-body PET imaging with a PD-L1–targeting tracer may have added value in detecting heterogenic expression of the drug target for anti–PD-1 therapy. Detection of low levels of PD-L1 expression may be predictive for nonresponding lesions that will require additional treatment. PET imaging of PD-L1 expression in non–small cell lung cancer, bladder cancer, and triple-negative breast cancer has shown that the intensity of the PET signal correlates with tumor response to ICI (*13**,**14*). The same approach could also be used to address the heterogeneity of metastatic tumor lesions in melanoma. This might especially be of importance for the characterization of brain lesions. PET imaging of PD-L1 expression may thus be a viable noninvasive diagnostic tool for lesion characterization and response prediction in metastatic melanoma patients.

This pilot study investigated the intensity and variability of PD-L1 expression using ^18^F-BMS986192 PET in metastatic tumor lesions of melanoma patients, including patients with brain metastases. As secondary objectives, we explored whether ^18^F-BMS986192 uptake was related to tumor response and whether enhanced tracer uptake in major organs was related to immune-related toxicity.

## MATERIALS AND METHODS

### Patient Population

Patients  18 y or older with stage IV metastatic melanoma who were eligible for treatment with ICI were recruited into the study. Patients with brain metastases were preferably included. Patients with suspected brain metastases could be treated with stereotactic radiotherapy before the start of the ICI therapy; however, these irradiated lesions were not included in the analyses. Other inclusion criteria were the presence of measurable disease according to RECIST, version 1.1; an Eastern Cooperative Oncology Group performance status of 0–1; and an adequate hematologic and end-organ function (*15*). The main exclusion criteria were preexisting autoimmune disease and treatment with immunosuppressive medication.

This study was approved by the Medical Ethical Committee (METc) of the University Medical Center Groningen (UMCG), delegated by the Central Committee on Research Involving Human Subjects, and was registered at ClinicalTrials.gov (ClinicalTrials.gov identifier NCT03520634) and METC 2016/646 (EUDRACT no. 2015-004920-67 with site-specific amendment 6). All patients provided written informed consent.

### Study Design

This single-center imaging study with the ^18^F-BMS986192 tracer was performed at the UMCG, The Netherlands. Patients received either nivolumab (anti–PD-1) or a combination of nivolumab and ipilimumab (anti–CTLA-4). The combination therapy consisted of 4 cycles of ipilimumab and nivolumab every 3 wk, followed by nivolumab monotherapy. Included patients were all treated with an ICI. The study intervention was a ^18^F-BMS986192 PET scan at baseline and 6 wk after treatment initiation.

### Production of ^18^F-BMS-986192

The azide precursor BMT-180478 and the adnectin BMT-192920, both required for the on-site preparation of the PET tracer ^18^F-BMS986192, were kindly provided by Brystol Myers Squibb (Supplemental Fig. 1; supplemental materials are available at http://jnm.snmjournals.org). The final PET tracer ^18^F-BMS-986192 was produced in a Good Manufacturing Practices–compliant automated synthesis module according to the previously published protocol (*16*). The radiochemical purity of ^18^F-BMS986192 was always more than 95%, and the molar activity always exceeded 3.000 GBq/mmol.

### ^18^F-BMS-986192 PET Acquisition and Analysis

Patients received an intravenous bolus injection of approximately 185 MBq (range, 182–192 MBq) of ^18^F-BMS986192. PET images were acquired 60 min after tracer injection on a Biograph mCT64 or mCT40 camera (Siemens Medical Systems). Whole-body PET scans (head to toe; 12 bed positions, 3 min per bed position) were acquired together with a low-dose CT scan. Vital signs (blood pressure and heart rate) were measured before, 10 min after the injection of ^18^F-BMS986192, and immediately after the PET/CT scan. Patients remained under observation for 120 min after tracer injection.

All PET scans were reconstructed according to the European Association of Nuclear Medicine (EANM) Research Ltd. (EARL) criteria and analyzed using Syngo.via VB30 software (Siemens) (*17*). For quantification of ^18^F-BMS986192 uptake, volumes of interest (VOIs) were manually drawn around the visible tumor lesions in the PET scan. For PET-negative lesions, the VOI was drawn around the tumor lesion on the CT or MRI scan that was manually aligned with the PET scan.

Tracer uptake in healthy tissues and lymphoid tissues (lung, thoracic aorta, spleen, liver, bone marrow, tonsils, parotid glands, thyroid, and axillary and inguinal lymph nodes) was measured to assess any toxicity-related increase in tracer uptake, using manually drawn VOIs. Tracer uptake was corrected for body weight and injected dose and expressed as SUVs (SUV_max_, SUV_mean_, and SUV_peak_ for tumor lesions and SUV_mean_ for normal tissues) consistent with EANM guidelines for ^18^F tracers in tumors (*18*). Because the SUV may be influenced by patient-specific characteristics, such as tracer metabolism and clearance, tumor-to-blood ratios (TBRs) were calculated using the VOI of the lesion and the thoracic aorta $\left(\text{TBR}\;=\;\frac{\text{Tumor}\;\;\text{SUV}}{\text{Blood-pool}\;\;\text{SUVmean}}\right)$ (*19*).

### Response Evaluation

Response to therapy was evaluated according to RECIST, version 1.1 (*15*). Contrast-enhanced chest–abdominal CT and gadolinium-enhanced MRI brain scans were obtained at baseline and week 12 as part of routine patient care.

Tumor lesions that were scanned on MR or CT at baseline but not at follow-up were excluded from response evaluation. To evaluate response per tumor lesion, the percentage change of the diameter at follow-up compared with baseline was calculated. Tumor lesions with a long-axis diameter smaller than 10 mm and lymph node lesions with a short-axis diameter smaller than 15 mm were excluded from response analysis.

Adverse events induced by the ICI treatment were assessed at each outpatient visit, using the National Cancer Institute Common Terminology Criteria for Adverse Events version 4.0 and reported in this study until the 12-wk tumor response evaluation.

### Statistical Analysis

Patients were evaluated for tracer biodistribution analysis if they underwent at least 1 ^18^F-BMS986192 PET scan. An assessment of the normality of data was performed using the Shapiro–Wilk test. Differences in tracer uptake between the baseline and on-treatment ^18^F-BMS986192 PET scans were analyzed using a paired *t* test. Correlations between parameters were calculated using the Spearman correlation test. *P* values of less than 0.05 were considered statistically significant. PET data are expressed as median with interquartile range for nonnormally distributed data and are expressed as mean with SD for normally distributed data.

Receiver-operating-characteristic (ROC) analyses were calculated to determine the ^18^F-BMS986192 uptake value that best differentiated between responding and nonresponding lesions. Statistical analyses were performed using GraphPad Prism 8.0.1 software for Microsoft Windows.

## RESULTS

A total of 10 patients were included in this study, 8 of whom underwent baseline ^18^F-BMS986192 PET/CT imaging before starting anti–PD-1 therapy (Table 1). In the remaining 2 patients, no PET scans could be obtained due to rapid clinical progression. In 4 patients, the on-treatment ^18^F-BMS986192 PET/CT scan was obtained between 41 and 55 d (mean, 42) after starting anti–PD-1 therapy. No ^18^F-BMS986192–related side effects occurred within the observation period of 120 min after tracer injection.

**TABLE 1.** Patient Characteristics

Characteristic	*n*
No. of patients included	10
No. of evaluable patients*	8
No. of lesions on PET	47
No. of evaluable lesions^†^	42
No. of lesions ≥ 10 mm at baseline (CT or MRI)	32
Median patient age at baseline (y)	62
No. of females (*n*)	3 (38)
No. of PET scans per patient (*n*)	
1	4 (50)
2	4 (50)
Immunotherapy regimen (*n*)	
Anti-CTLA4 + Anti-PD-1	5 (62)
Anti-PD1	3 (38)
No. of patients by treatment response (RECIST, version 1.1) (*n*)	
Complete response	0 (0)
Partial response	2 (25)
Stable disease	2 (25)
Progressive disease	4 (50)

* Including 6 patients with brain metastases.

^†^Lesions that were detected at baseline and follow-up by MR or CT imaging.

Data in parentheses are percentages.

### Biodistribution of ^18^F-BMS986192

**FIGURE 1.**
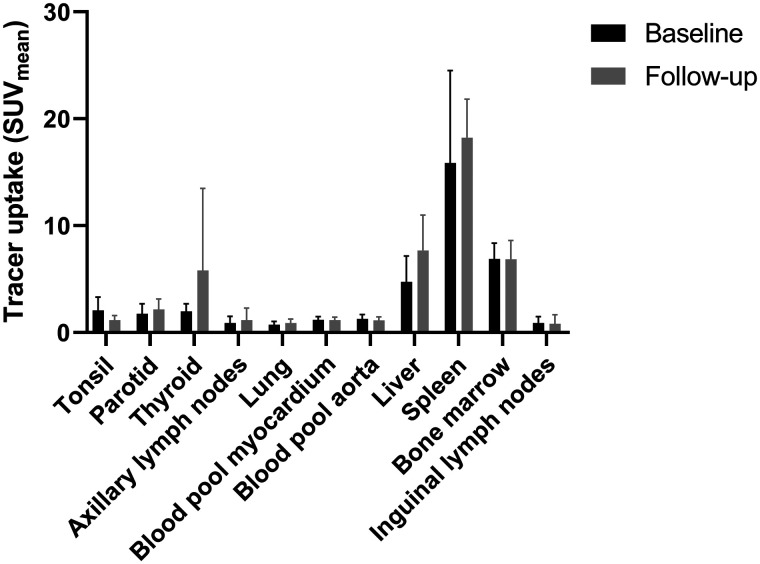
Biodistribution of ^18^F-BMS986192 at baseline (*n* = 8) and follow-up (*n* = 4). In the patients with follow-up scans, ^18^F-BMS986192 uptake in the tonsil was significantly higher at baseline than at follow-up (Wilcoxon signed rank test, *P* = 0.002). There were no other differences in ^18^F-BMS986192 uptake between baseline and follow-up.

The biodistribution of ^18^F-BMS986192 was evaluated in healthy tissues at baseline. High tracer uptake at baseline was observed in the spleen (SUV_mean_, 15.9 ± 8.7), bone marrow (SUV_mean_, 6.9 ± 1.5), and liver (SUV_mean_, 4.7 ± 2.4). Patients with a follow-up scan (*n* = 4) did not show significant changes in uptake in healthy tissues compared with the baseline PET scan, except for tonsil uptake, which was significantly higher at baseline than at follow-up (Wilcoxon signed-rank test, *P* = 0.002) (Fig. 1).

### Variability in ^18^F-BMS986192 Uptake in Metastatic Lesions

**FIGURE 2.**
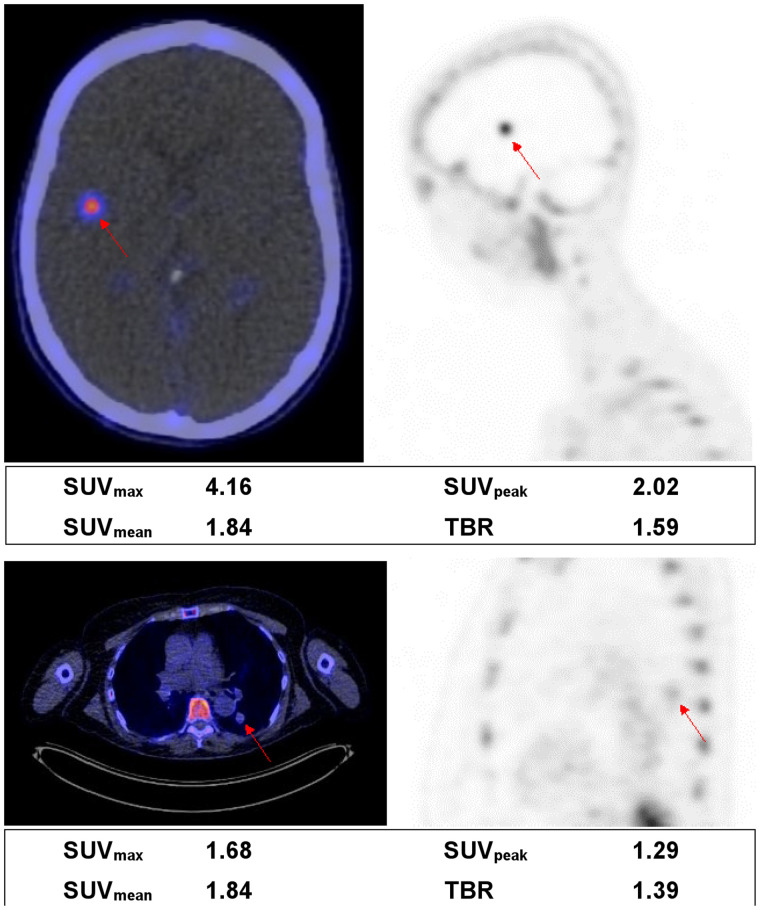
Examples of a brain lesion (top) and a lung lesion (bottom). Fused PET/CT transverse images (left) and PET sagittal images (right) are shown. Semiquantitative measurements are reported underneath images. TBR is calculated as lesion SUV_peak_ divided by SUV_mean_ in aortic blood pool.

**FIGURE 3.**
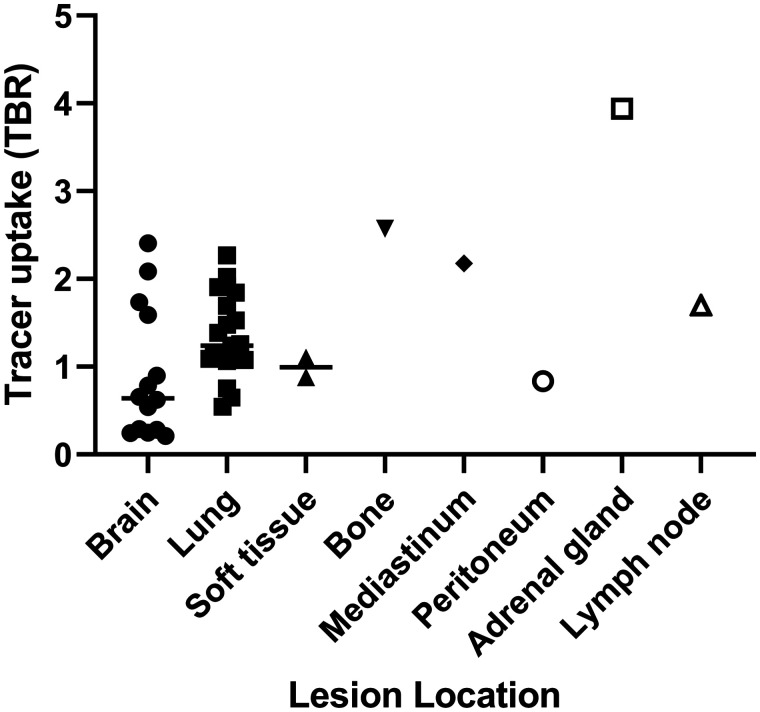
Baseline ^18^F-BMS986192 tracer uptake measured as TBR derived from SUV_peak_ of lesion and SUV_mean_ of aortic blood pool. Median group value is reported as a line. No significant difference was found between mean values of brain and lung lesions (Mann–Whitney *U* test, *P* = 0.06). Analysis included 42 tumor lesions (21 lung; 14 brain; 2 soft-tissue; and 1 each in bone, mediastinum, peritoneum, adrenal gland, and lymph node lesions) in 8 patients.

Of the 42 evaluable tumor lesions, 21 were located in the lungs; 14 in the brain; 2 in soft tissue; and 1 each in bone, mediastinum, peritoneum, adrenal gland, and lymph node. Examples of PET images of a brain and lung lesion are depicted in Figure 2. The variability in uptake is shown in Figure 3 and Supplemental Figures 2 and 3. No significant differences were observed in ^18^F-BMS986192 uptake between lung and brain lesions (*P* = 0.06). One bone lesion was excluded from analyses because its VOI could not be sufficiently separated from the high bone marrow uptake nearby, resulting in an uptake value 6 SDs higher than the other metastatic lesions.

### Association Between Baseline Tumor Uptake and Response to Therapy

After the exclusion of small tumor lesions (median diameter, 5 mm; range, 4–9 mm), 32 lesions (8 brain lesions) remained for response evaluation. The percentage change in lesion diameter was not associated with the baseline tracer uptake parameters SUV_max_ (r = −0.15, *P* = 0.42), SUV_mean_ (r = −0.17, *P* = 0.34), or SUV_peak_ (r = −0.18, *P* = 0.31).

**FIGURE 4.**
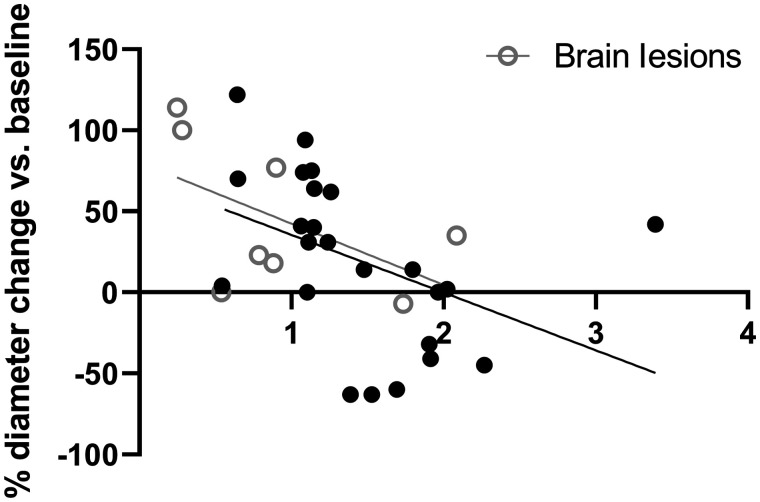
Association of TBR, derived from SUV_peak_ of lesion and SUV_mean_ of aortic blood pool, and relative (%) diameter change of lesions (Spearman rank, r = −0.43, *P* = 0.014). From all 8 patients, each circle represents 1 lesion. Only lesions larger than 10 mm at baseline were included in this analysis (*n* = 32). ○ = brain lesions (*n* = 8).

When correcting for differences in tracer availability, using the tracer uptake in the blood pool as reference, we observed a significant correlation between baseline tumor-to-blood ratio and change in tumor lesion size (Fig. 4). This negative association was seen when calculating a TBR from the SUV_max_ (r = −0.43, *P* = 0.014), SUV_mean_ (r = −0.44, *P* = 0.012), or SUV_peak_ (r = −0.49, *P* = 0.005) of the tumor and SUV_mean_ of the blood pool. The same negative association was observed when only analyzing the intracerebral (brain) lesions, but this association was statistically not significant (r = −0.54, *P* = 0.17).

### Discrimination of Responding and Nonresponding Tumors

**FIGURE 5.**
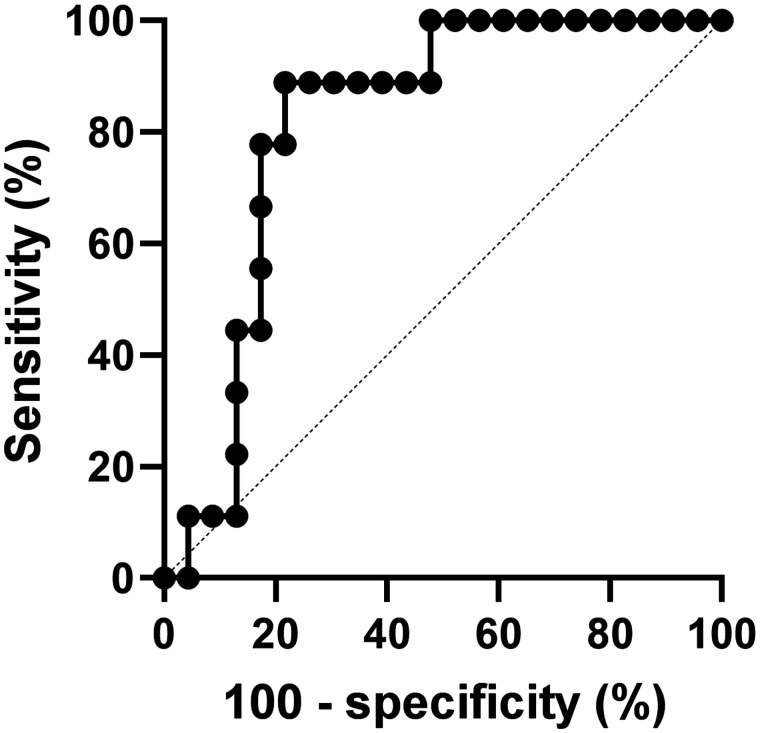
ROC curve of TBR of lesion, showing sensitivity and specificity for discriminating between tumors increasing or decreasing in diameter. Area under the curve is 0.82 (*P* = 0.006).

ROC analysis was performed to assess the optimal tracer uptake value for predicting an ICI-induced reduction in tumor diameter. When a TBR derived from SUV_peak_ of the lesion and SUV_mean_ of the aortic blood pool was used, ROC analysis resulted in an area under the curve of 0.82 (*P* = 0.006). A TBR cutoff value of 1.3 resulted in a sensitivity of 89% (95% CI, 57%–99%) and a specificity of 78% (95% CI, 58%–90%) for discriminating a tumor decreasing in size from a tumor increasing in size (Fig. 5).

### Follow-up ^18^F-BMS986192 Scan and Response to Therapy

**FIGURE 6.**
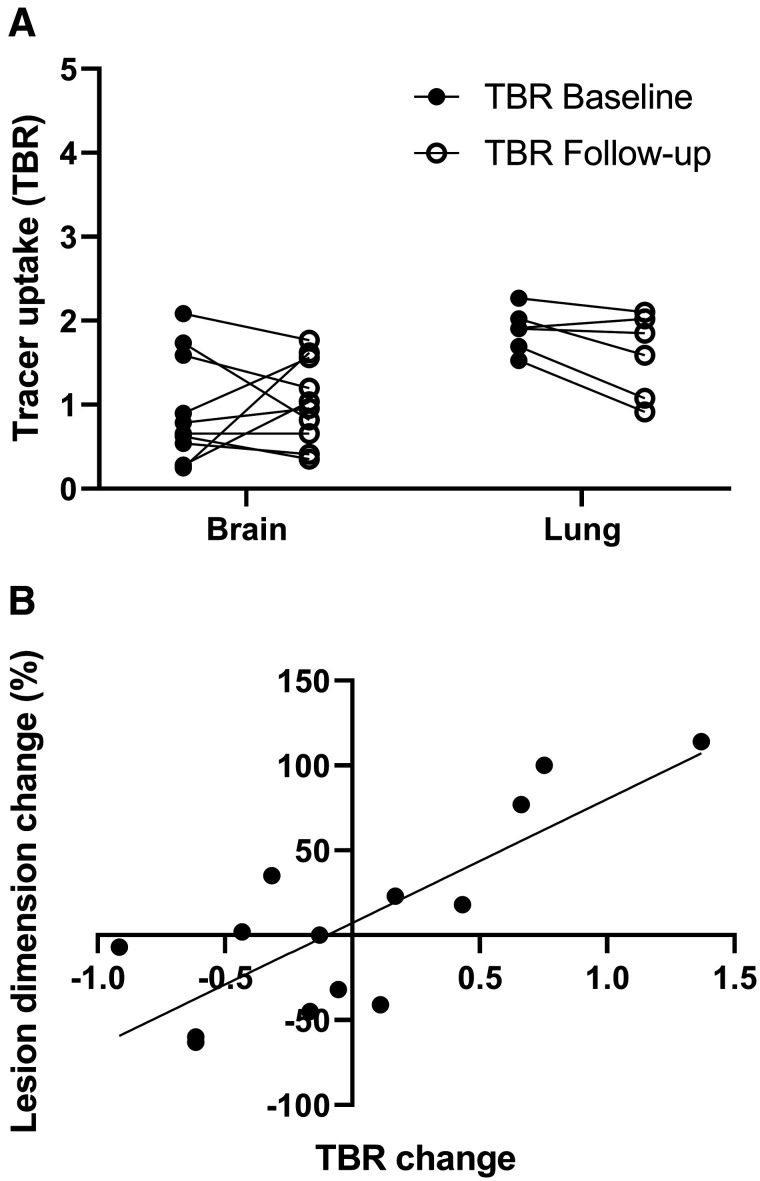
(A) ^18^F-BMS986192 uptake for brain and lung lesions at baseline and follow-up (4 patients, 16 lesions). Tracer uptake is reported as TBR. Significant differences in tracer uptake were observed between brain and lung lesions, both at baseline and at follow-up (Wilcoxon signed rank test, *P* = 0.004 and 0.05, respectively). (B) Association between change in lesion size and change in TBR of follow-up ^18^F-BMS986192 scan compared with baseline (TBR_follow-up_^–^ TBR_baseline_) (4 patients, 14 lesions). Significant positive correlation was found between increasing TBR and increasing lesion diameter (Spearman rank, *r* = 0.79, *P* = 0.0007).

Four patients (50%) with a total of 19 metastases underwent a follow-up ^18^F-BMS986192 scan at 6 wk. A paired *t* test revealed no differences in tumor ^18^F-BMS986192 uptake between baseline and follow-up scans. Figure 6A shows the individual differences in tumor ^18^F-BMS986192 uptake between baseline and follow-up for the brain and lung lesions in these 4 patients. ^18^F-BMS986192 uptake was significantly lower in the brain lesions than the lung lesions, both at baseline and on-treatment (*P* = 0.004 and 0.05, respectively). No differences were observed in the mean lesion size of these brain and lung lesions.

Fourteen of the 19 metastases on follow-up scans were larger than 10 mm at baseline. A significant correlation between the change in ^18^F-BMS986192 uptake (TBR) and the change in tumor size between the baseline and follow-up was observed (*r* = 0.79, *P* = 0.0007; Fig. 6B).

### Assessment of Toxicity: Increased Uptake of ^18^F-BMS986192

**FIGURE 7.**
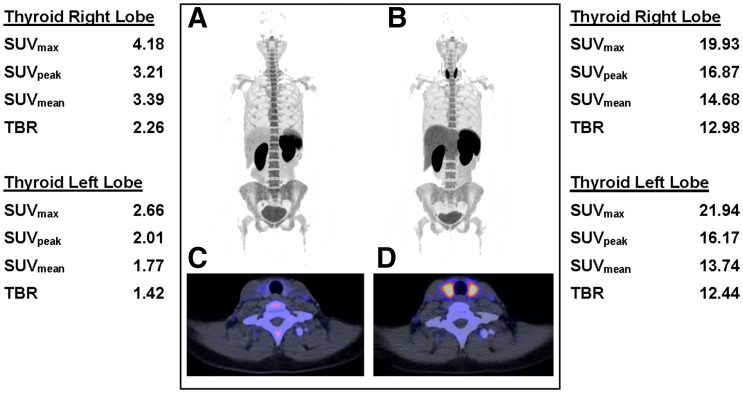
^18^F-BMS986192 -L1 PET/CT scans of 2 patients who developed ICI-related toxicity. (A–D) ^18^F-BMS986192 scans of patient who developed ICI-related thyroiditis with increased uptake on treatment (B and D) compared with baseline (A and C). Semiquantitative measurements are shown for baseline scan (left) and the follow-up scan (right). TBR was calculated by dividing thyroid SUV_peak_ by SUV_mean_ in aortic blood pool.

Follow-up ^18^F-BMS986192 scans were also evaluated for ICI-related toxicity. One patient developed ICI-related hyperthyroidism, another patient colitis. The follow-up PET scan of the patient with ICI-related hyperthyroidism showed increased uptake in the thyroid (Fig. 7). This diagnosis was substantiated by blood thyroid-stimulating hormone levels of 0.007 mU/L (normal, 0.5–4 mU/L) and free T4 levels of 37.2 pmol/L (normal, 11–19.5 pmol/L). The patient who developed ICI-related colitis 1 d after the PET scan showed a different bowel uptake pattern at the second scan compared with the first scan, but solely based on the PET scan a definitive diagnosis of colitis could not be settled.

## DISCUSSION

To our knowledge, this is the first study to investigate ^18^F-BMS986192 PET imaging before and during ICI therapy in patients with metastatic melanoma, including patients with brain metastases. We found heterogeneous uptake of ^18^F-BMS986192 in metastatic lesions at all locations, which is in line with a heterogeneous response to ICI therapy that is often observed in melanoma patients. Interestingly, we observed that high baseline ^18^F-BMS986192 uptake in lesions, corrected for blood-pool uptake, correlated with response to ICI treatment. The same trend was found for brain lesions, which is an important finding because nonresponding brain lesions often require additional treatment. Additionally, increased ^18^F-BMS-986192 uptake in tumor lesions at the follow-up ^18^F-BMS986192 PET scan at week 6 was correlated with tumor progression at 12 wk.

Within a single patient with metastatic melanoma, different metastatic lesions may respond variably to ICI therapy. This variability in response to ICI is both in terms of the change in the lesion volume and the time it takes a lesion to show response (*20*). Because of this variability in response to ICI, additional local treatment is often necessary on nonresponding metastatic melanoma lesions (*10*). High immunohistochemistry PD-L1 expression in a metastatic lesion is associated with a favorable response to ICI therapy. Importantly, metastatic melanoma patients with low PD-L1 expression in a lesion may also respond to ICI therapy (*12*). Therefore, immunohistochemistry PD-L1 expression on biopsies is not used in clinical practice to predict a patient’s response to ICI therapy.

Discriminating between the different types of cells that may express PD-L1 in a metastatic melanoma lesion, such as malignant melanocytes, monocytes, and macrophages, may be important for ICI therapy response prediction (*21*). For example, pretreatment PD-L1 expression on macrophages was associated with favorable response to ICI therapy, whereas pretreatment PD-L1 expression on malignant melanocytes did not show any association with response (*22*). Although whole-body PD-L1 PET cannot differentiate between the PD-L1 expression of different cellular compositions, it may be useful to visualize PD-L1 expression in different lesions and predicting response per tumor lesion to ICI. Moreover, lesions with low PD-L1 expression on PET may indicate those lesions that need additional local treatment.

In our study, high ^18^F-BMS986192 uptake was observed in lymphoid tissues, such as the spleen and bone marrow. This is suggestive for PD-L1 targeting of the tracer ^18^F-BMS986192, as these organs contain high levels of PD-L1–expressing immune cells. Additionally, the follow-up ^18^F-BMS986192 PET scan showed an increased PD-L1 uptake in 2 patients with ICI-induced toxicity (thyroiditis and colitis), which may be a sign of inflammation with an increase in PD-L1–expressing immune cells.

The correlation of PD-L1 uptake in tumor lesions at baseline with response to treatment found in our study is comparable with a study in non–small lung cancer. That study demonstrated that both ^18^F-BMS-986192 (PD-L1) and ^89^Zr-nivolumab (PD-1) PET imaging could predict response on a lesion level (*13*). In addition, in our study the second ^18^F-BMS986192 scan during ICI treatment at week 6 performed in 4 patients found an increased ^18^F-BMS986192 uptake correlating significantly with increased tumor size at follow-up at 12 wk. This positive correlation can be explained by the fact that responding lesions have fewer PD-L1–expressing tumor cells, resulting in a reduction in ^18^F-BMS986192 uptake. On the other hand, a higher tracer uptake at week 6 in nonresponding tumors at week 12 might theoretically be due to either a higher cell density or an increased expression of PD-L1 per cell. PET cannot discriminate between these options. It is described that high PD L1–expressing tumor cells is related to a higher density of PD-1 tumor–associated T cells (*23*). Therefore, a delayed immune influx in PD-L1–upregulated tumor lesions at week 6 could be responsible for pseudoprogression at week 12. Unfortunately, our pilot study was unable to record any late response to immune checkpoint inhibition therapy as by that time these patients had already switched to another form of treatment. Moreover, technical aspects may have contributed to the correlation between the change in tumor size and the change in tracer uptake. A reduction in tumor size could also lead to a stronger partial-volume effect (spill-out effect), which would also lead to the reduction in ^18^F-BMS986192 uptake.

Protein-based PET tracers targeting PD-L1 do not accumulate in normal brain tissue (*13**,**14*), because proteins usually cannot cross the intact blood–brain barrier. The inability of large proteins to enter the brain could be part of the cause of the mixed response of brain metastases to treatment with ICI. Brain metastases can disrupt the blood–brain barrier, but this is not always the case. Niemeijer et al. demonstrated that uptake of the protein-based PD-L1 tracers ^18^F-BMS-986192 and ^89^Zr-DFO-nivolumab was observed in some, but not all, untreated brain metastases in 2 non–small lung cancer patients (*13*). In our study, the ^18^F-BMS986192 uptake in brain metastases was also heterogeneous. Moreover, ^18^F-BMS986192 uptake in brain metastases showed a relation with response, although this correlation was not statistically significant, but this may be due to the sample size.

In this study, a ^18^F-labeled adnectin was used to target PD-L1 protein expression. In contrast to PET with the ^89^Zr-linked antibody tracer ^89^Zr-DFO-atezolizumab, ^18^F-BMS986192 PET provides the opportunity to acquire the PET scan 1 h after tracer injection, and thus the examination can be completed within a single visit, which is highly convenient for the patient. Imaging on the same day as tracer injection is especially important for patients with brain metastases, where it is often essential to start treatment without delay. In addition, ^18^F-BMS986192 PET exposes the patients to a much lower radiation dose, allowing the acquisition of multiple ^18^F-BMS986192 PET examinations in the same patients within a short time frame. Moreover, it should be emphasized that the tumor accumulation of ^18^F-BMS986192 and most ^89^Zr-labeled antibody tracers is based on slightly different mechanisms. ^18^F-BMS986192 uptake in the tumor is the result of reversible receptor binding and thus reflect receptor expression (*24*). The uptake of ^89^Zr-labeled antibody tracers, on the other hand, is the result of receptor binding, followed by receptor internalization, and thus represents receptor turnover, more than receptor expression. The lower radiation burden, the fast tracer kinetics, and the uptake mechanism of ^18^F-BMS986192 make this tracer better suited for pharmacokinetics and pharmacodynamics studies than the ^89^Zr-labeled antibody tracers.

## CONCLUSION

PET imaging with ^18^F-BMS986192 PET shows heterogeneous PD-L1 expression in brain and lung metastases of melanoma patients. The ^18^F-BMS986192 tumor uptake correlates with response on a per-lesion basis, with the same trend found in both intracerebral and extracerebral lesions. ICI-related toxicity can be detected at an early stage, before clinical symptoms appear. The preliminary results of this pilot study warrant further evaluation of ^18^F-BMS986192 PET as a noninvasive imaging tool for assessment of PD-L1 expression in (brain) tumor lesions and prediction of ICI therapy response.

## Notes

KEY POINTS **QUESTION:** Is variability of PD-L1 PET tracer uptake in metastatic melanoma lesions related to tumor response on ICIs, particularly in brain metastases? **PERTINENT FINDINGS:** In this pilot study, ^18^F-BMS986192 PET showed heterogeneous uptake in intra- and extracerebral metastatic lesions in melanoma patients. Baseline ^18^F-BMS986192 uptake was able to predict an ICI-induced reduction in lesion volume. **IMPLICATIONS FOR PATIENT CARE:** In melanoma patients, PD-L1 PET imaging may identify which metastatic lesions require local treatment in addition to ICIs.

## References

[1] Ascierto PA Long GV Robert C. survival outcomes in patients with previously untreated BRAF wild-type advanced melanoma treated with nivolumab therapy: three-year follow-up of a randomized phase 3 trial. JAMA Oncol. 2019;5:187–194. 30422243 10.1001/jamaoncol.2018.4514 PMC6439558

[2] Robert C Long GV Brady B. Nivolumab in previously untreated melanoma without BRAF mutation. N Engl J Med. 2015;372:320–330. 25399552 10.1056/NEJMoa1412082

[3] Topalian SL Sznol M McDermott DF. Survival, durable tumor remission, and long-term safety in patients with advanced melanoma receiving nivolumab. J Clin Oncol. 2014;32:1020–1030. 24590637 10.1200/JCO.2013.53.0105 PMC4811023

[4] Weber JS D’Angelo SP Minor D. Nivolumab versus chemotherapy in patients with advanced melanoma who progressed after anti-CTLA-4 treatment (CheckMate 037): a randomised, controlled, open-label, phase 3 trial. Lancet Oncol. 2015;16:375–384. 25795410 10.1016/S1470-2045(15)70076-8

[5] Schachter J Ribas A Long GV. Pembrolizumab versus ipilimumab for advanced melanoma: final overall survival results of a multicentre, randomised, open-label phase 3 study (KEYNOTE-006). Lancet. 2017;390:1853–1862. 28822576 10.1016/S0140-6736(17)31601-X

[6] Ascierto PA Del Vecchio M Robert C. Ipilimumab 10 mg/kg versus ipilimumab 3 mg/kg in patients with unresectable or metastatic melanoma: a randomised, double-blind, multicentre, phase 3 trial. Lancet Oncol. 2017;18:611–622. 28359784 10.1016/S1470-2045(17)30231-0

[7] Robert C Thomas L Bondarenko I. Ipilimumab plus dacarbazine for previously untreated metastatic melanoma. N Engl J Med. 2011;364:2517–2526. 21639810 10.1056/NEJMoa1104621

[8] Hodi FS Chiarion-Sileni V Gonzalez R. Nivolumab plus ipilimumab or nivolumab alone versus ipilimumab alone in advanced melanoma (CheckMate 067): 4-year outcomes of a multicentre, randomised, phase 3 trial. Lancet Oncol. 2018;19:1480–1492. 30361170 10.1016/S1470-2045(18)30700-9

[9] Wolchok JD Hoos A O’Day S. Guidelines for the evaluation of immune therapy activity in solid tumors: Immune-related response criteria. Clin Cancer Res. 2009;15:7412–7420. 19934295 10.1158/1078-0432.CCR-09-1624

[10] Blankenstein SA Aarts MJB van den Berkmortel FWPJ. Surgery for unresectable stage IIIC and IV melanoma in the era of new systemic therapy. Cancers (Basel). 2020;12:1176. 10.3390/cancers12051176 PMC7281176 32392717

[11] Noh T Walbert T. Brain metastasis: clinical manifestations, symptom management, and palliative care. Handb Clin Neurol. 2018;149:75–88. 29307363 10.1016/B978-0-12-811161-1.00006-2

[12] Santos-Briz A Cañueto J del Carmen S. Value of PD-L1, PD-1, and CTLA-4 expression in the clinical practice as predictors of response to nivolumab and ipilimumab in monotherapy in patients with advanced stage melanoma. Am J Dermatopathol. 2021;43:423–428. 33395045 10.1097/DAD.0000000000001856

[13] Niemeijer AN Leung D Huisman MC. Whole body PD-1 and PD-L1 positron emission tomography in patients with non-small-cell lung cancer. Nat Commun. 2018;9:4664. 30405135 10.1038/s41467-018-07131-y PMC6220188

[14] Bensch F van der Veen EL Lub-de Hooge MN. ^89^Zr-atezolizumab imaging as a non-invasive approach to assess clinical response to PD-L1 blockade in cancer. Nat Med. 2018;24:1852–1858. 30478423 10.1038/s41591-018-0255-8

[15] Eisenhauer EA Therasse P Bogaerts J. New response evaluation criteria in solid tumours: Revised RECIST guideline (version 1.1). Eur J Cancer. 2009;45:228–247. 19097774 10.1016/j.ejca.2008.10.026

[16] Donnelly DJ Adam Smith R Morin P. Synthesis and biologic evaluation of a novel ^18^F-labeled adnectin as a PET radioligand for imaging PD-L1 expression. J Nucl Med. 2018;59:529–535. 29025984 10.2967/jnumed.117.199596

[17] Kaalep A Sera T Oyen W. EANM/EARL FDG-PET/CT accreditation - summary results from the first 200 accredited imaging systems. Eur J Nucl Med Mol Imaging. 2018;45:412–422. 29192365 10.1007/s00259-017-3853-7 PMC5787222

[18] Boellaard R Delgado-Bolton R Oyen WJGG. FDG PET/CT: EANM procedure guidelines for tumour imaging: version 2.0. Eur J Nucl Med Mol Imaging. 2015;42:328–354. 25452219 10.1007/s00259-014-2961-x PMC4315529

[19] Hofheinz F Bütof R Apostolova I. An investigation of the relation between tumor-to-liver ratio (TLR) and tumor-to-blood standard uptake ratio (SUR) in oncological FDG PET. EJNMMI Res. 2016;6:19. 26936768 10.1186/s13550-016-0174-y PMC4775714

[20] Persigehl T Lennartz S Schwartz LH. IRECIST: how to do it. Cancer Imaging. 2020;20:2. 31900236 10.1186/s40644-019-0281-x PMC6942293

[21] Tang F Zheng P. Tumor cells versus host immune cells: whose PD-L1 contributes to PD-1/PD-L1 blockade mediated cancer immunotherapy? Cell Biosci. 2018;8:34. 29744030 10.1186/s13578-018-0232-4 PMC5930423

[22] Toki MI Merritt CR Wong PF. High-plex predictive marker discovery for melanoma immunotherapy-treated patients using digital spatial profiling. Clin Cancer Res. 2019;25:5503–5512. 31189645 10.1158/1078-0432.CCR-19-0104 PMC6744974

[23] Kandl TJ Sagiv O Curry JL. High expression of PD-1 and PD-L1 in ocular adnexal sebaceous carcinoma. Oncoimmunology. 2018;7:e1475874. 30228943 10.1080/2162402X.2018.1475874 PMC6140585

[24] Stutvoet TS van der Veen EL Kol A. Molecular imaging of PD-L1 expression and dynamics with the adnectin-based PET tracer ^18^ F-BMS-986192. J Nucl Med. 2020;61:1839–1844. 32358092 10.2967/jnumed.119.241364

